# Methylphenidate for Attention-Deficit and Hyperactivity Disorder in Adult Patients With Substance Use Disorders: Good Clinical Practice

**DOI:** 10.3389/fpsyt.2020.540837

**Published:** 2021-01-26

**Authors:** Sunsha Chamakalayil, Johannes Strasser, Marc Vogel, Serge Brand, Marc Walter, Kenneth M. Dürsteler

**Affiliations:** ^1^Department of Addictive Disorders, Psychiatric University Clinic Basel, Basel, Switzerland; ^2^Department of Addictive Disorders, Psychiatric Services Thurgau, Münsterlingen, Switzerland; ^3^Center for Affective, Stress and Sleep Disorders (ZASS), Psychiatric University Hospital Basel, Basel, Switzerland; ^4^Department of Clinical Research, University of Basel, Basel, Switzerland; ^5^Division of Sport and Psychosocial Health, Department of Sport, Exercise, and Health, University of Basel, Basel, Switzerland; ^6^Substance Abuse Prevention Research Center and Sleep Disorders Research Center, Kermanshah University of Medical Sciences, Kermanshah, Iran; ^7^School of Medicine, Tehran University of Medical Sciences (TUMS), Tehrren, Iran; ^8^Department for Psychiatry, Psychotherapy and Psychosomatic, Psychiatric Hospital, University of Zurich, Zurich, Switzerland

**Keywords:** ADHD, methylphenidate, substance use disorder, diagnosis, therapy, misuse

## Abstract

Attention-deficit and hyperactivity disorder (ADHD) is a widespread neurodevelopmental disorder in children and adolescents, persisting into adulthood in a majority of them. ADHD and substance use disorders (SUDs) commonly co-occur in the clinical adult population. The higher-than-normal prevalence rates of SUDs in people with ADHD indicate increased risk for developing SUD. This narrative review deals with the question of whether or not adults with both disorders should be treated with methylphenidate (MPH), addressing specific issues surrounding this form of treatment. MPH is considered as first-line pharmacotherapy for ADHD. However, because of its stimulant-like reinforcing properties, MPH has a significant addictive potential to which persons with SUDs are especially susceptible. Appropriate treatment is therefore complex. Because of concerns about misuse and diversion of MPH medication, clinicians may be reluctant to use MPH to manage ADHD symptoms in these patients. However, it is essential to diagnose and treat ADHD adequately as appropriate therapy reduces the impairments, as well as the risk of developing comorbid disorders and poor treatment response. MPH should not be deprived of these patients because of the risk for misuse, especially as several strategies can be applied to minimize this risk. To conclude, carefully applied guideline-based diagnostics to clarify the potential presence of ADHD as well as a responsible prescription practice in a well-defined therapeutic setting with reliable monitoring of medication intake and regular consultations are essential conditions for a safe and proficient MPH treatment of ADHD in patients with SUD.

## Introduction

### Attention-Deficit and Hyperactivity Disorder

The prevalence of attention-deficit and hyperactivity disorder (ADHD) in children and adolescents is estimated to be between 2 and 7% (1), whereby some studies found prevalence rates up to 16% in certain age groups (2). The prevalence rates vary depending on the underlying classification system, and boys are diagnosed about two to three times more often with ADHD than girls (3–5). Already during early infancy, people with ADHD often exhibit signs, such as frequent crying, feeding and sleeping problems, restless sleep, and excessive unrest (6–8). Subsequently, the key features of ADHD manifest inattention, impulsivity, and hyperactivity.

ADHD is a multifactorial, clinically heterogeneous neurodevelopmental disorder (9) caused by interplay between genetic and environmental factors (10). It is assumed to result from suboptimal dopamine levels in the synaptic cleft due to overexpression of the presynaptic dopamine transporter (DAT) (11). Numerous studies have proven a familial aggregation of ADHD (5, 12). Environmental risk factors are, e.g., prenatal exposure to alcohol and tobacco, premature birth, critical birth circumstances, and incongruities in parent–child interactions, such as difficulties in feeding the infant, etc. (2, 12). Based on genetic predisposition and psychosocial risks, ADHD leads, among others, to neurocognitive/behavioral problems due to neurobiological dysregulation. These problems manifest mainly in the area of attention and executive functions (2, 13). People with ADHD show deficits in attention intensity and selectivity, in executive inhibitory control (of, e.g., motor action or prepotent responses) and therefore in self-regulation, as well as in memory functions, especially in short-term/working memory (2). They therefore struggle with continuous vigilance and attention, are easily distracted by internal and/or external stimuli, experience themselves as “forgetful,” and express difficulty in self-organizing and performing their daily routines. Other neurocognitive performances are usually not affected.

The long-prevailing view that ADHD is a disorder of childhood and adolescence, phasing out in adulthood, has been refuted (14–16). Even in adulthood ADHD is a disorder that leads to several mental problems and serious social issues (17). According to current knowledge, in 60% of the affected individuals, some or even all symptoms of the disorder will persist into adulthood. Depending on the study, the prevalence of ADHD in adults ranges from 1 to 4% (18, 19), with less pronounced gender differences than in children and adolescents (20).

Although the neurobiological functional deficits remain the central problem for all age groups, the severity and course of the disorder in adults are extremely heterogeneous (19). Impairments in everyday life are often more multifaceted than in children, whereas hyperactivity fades often into the background. Issues resulting from attention deficits are much more characteristic in adults so that difficulties in performing daily routine tasks often lead to severe problems at home, at work, and in social relationships (13). Moreover, the suboptimal regulation of affect and impulse control often impair social interactions, increasing the psychological strain of the affected people and their living environment and favoring the occurrence of other mental disorders.

This article briefly reviews existing literature on methylphenidate (MPH) treatment of adult ADHD in persons with substance use disorders (SUDs), especially with opioid use disorder (OUD). It takes up on specific problems, such as misuse and diversion of MPH, which are associated with MPH therapy in these patients. It also aims to give recommendations on diagnostics, therapy, and treatment settings, when dealing with these issues.

## Methods

This narrative review is predominantly based on a MEDLINE database search. Additionally, importance was given to include guidelines for clinical practice, such as NICE guidelines (21) or consensus statements of experts (22, 23), in order to address specific issues, which arise during the treatment of patients with ADHD as well as SUD.

## Results

### ADHD and SUDs

Co-occurring ADHD and SUDs are routinely encountered in clinical settings (24). Studies have shown that the prevalence rates of SUDs are two to four times higher in people with ADHD than in the normal population (25, 26). In clinical samples, one-fifth of all alcohol-dependent and up to one-third of all cocaine-dependent patients meet criteria for adult ADHD (27, 28). In patients receiving opioid agonist treatment (OAT), the prevalence rates range between 20 and 25% (17, 29, 30). Overall, the prevalence rates of adult ADHD in SUD treatment settings range up to 24% (30). Both the severity and persistence of ADHD symptoms seem to influence the risk of developing an SUD and to reduce the effectiveness of treatment (31).

The relationship between ADHD and SUD is complex. As psychoactive substances reduce, imitate, or aggravate the symptoms of ADHD, differentiating between both disorders is challenging, which impedes the research in this field (32, 33). There is an ongoing debate whether common genetic factors or the attempt of self-medication causes the increased vulnerability for SUDs in persons with ADHD (15, 34). However, there seems to be no preference for any specific SUD (28, 35, 36).

Studies exploring common neuronal pathways in ADHD and SUDs indicate that anomalies in circuits related to reward processing, especially delayed reward processing (37, 38), might be present in both disorders (39, 40). Circuits related to reward processing are the mesolimbic and mesocortical pathways. The mesolimbic pathway involves mainly dopaminergic projections from the ventral tegmental area (VTA) in the midbrain through the median forebrain bundle to the nucleus accumbens and the limbic system with amygdala, hypothalamus, and hippocampus (41). Particularly, the mesoaccumbens dopamine pathway projecting from the VTA to the nucleus accumbens is associated with reward and motivation (42). The nucleus accumbens, a major component of the ventral striatum, is associated with the outcome evaluation of the reward (43). The mesocortical pathway involves mainly dopaminergic projections from the VTA to the prefrontal cortex (PFC) (44) and the anterior cingulate cortex (ACC). These structures are involved in executive functioning, such as decision-making or impulse control (45). In ADHD and SUDs, similar deficits in dopamine activity in these regions were found (39, 46). Positron emission tomography studies showed that ADHD and SUD seem to be associated with a reduced D_2_/D_3_ receptor availability in the midbrain, caudate nucleus (part of dorsal striatum), and hypothalamus (39, 42, 46). DAT binding was diffusely decreased across nucleus accumbens, midbrain, left caudate nucleus, and hypothalamus (39). PFC is also interconnected to the caudate nucleus, which plays an important role in procedural learning and inhibitory control of action (47). Functional magnetic resonance imaging studies suggest a reduced activation in frontostriatal brain regions (46). Furthermore, underactivity in the ACC and orbitofrontal cortex was associated with both ADHD and SUD symptoms (39). A meta-analysis reported reduced ACC gray matter volumes in adults with ADHD (48). Also, smaller right putamen and right cerebellum gray matter volumes were reported in adult ADHD (49). In people with ADHD and cocaine use disorder, Wingen et al. reported smaller occipital cortical gray matter volumes and reduced volumes in the putamen compared to people with ADHD (49).

### Diagnostics and Therapy

Appropriate diagnostic workup and therapy of adult ADHD is important and necessary. First, the disorder is common but often remains unrecognized (22). Second, it leads to severe mental health and social impairments. Third, ADHD increases the risk of developing many other mental disorders, especially SUDs, affective disorders, anxiety disorders, and personality disorders (19). Fourth, the syndrome is well-treatable, and therapy reduces the risk for comorbid disorders, psychosocial problems, and treatment failure due to poor treatment response (2, 50).

Compared to children and adolescents, there is a great backlog in the diagnostics and therapy of ADHD in adults (32). To diagnose ADHD in adults, it is essential that the core symptoms started before the age of 7 [or 12 according to *Diagnostic and Statistical Manual of Mental Disorders* (*DSM-5*)] and are, at least in part, still present in adulthood. The diagnostic process should be performed, if possible, under abstinent conditions. Also, the individuals in OAT should be well-adjusted to the medication (51), so that the present symptoms can be clarified in-depth and assessed reliably according to valid diagnostic criteria [*DSM-IV*/*DSM-5*/*International Classification of Diseases, 10th Revision* (*ICD-10*)].

To ensure a careful and comprehensive evaluation for possible ADHD, a guideline-based diagnostic procedure is recommended in current clinical practice (21). Although *ICD-10* offers the possibility to code the presence of ADHD, the criteria for adults are not explicitly mentioned. Diagnosis based on guidelines includes the developmental psychopathology of ADHD by stating the collection of retrospective symptoms as an integral part of the diagnosis.

According to NICE guidelines (21), the diagnostic evaluation consists of multiple phases with different aims. It requires patience, time, and the inclusion of several methods and sources of information. For diagnosis and treatment, it is always important to reconstruct the individual course of the disorder as precisely as possible. The purpose of a thorough and detailed psychiatric history is to identify individual problems considering possible comorbid mental disorders, the history of development of the individuals, and their family of origin. Already the history may indicate the presence of ADHD. The clinical impression is very important, but it is not sufficient to diagnose ADHD. For differential diagnosis and for assessing other mental disorders apart from the clinical impression, the use of structured instruments (e.g., Structured Clinical Interview for *DSM-IV*/*DSM-5*) is recommended. In patients with SUD, a thorough assessment of the current SUDs is essential to evaluate the influence of substance use on ADHD symptoms (52).

The next step is to collect a complete medical history and to perform a physical examination to exclude organic causes, such as thyroid disease, seizure disorder, or sleep disorder. To assess former and current symptoms, parents/siblings, and other important persons of trust should be interviewed. Validated structured interviews, such as Conners' Adult ADHD Diagnostic Interview for *DSM-IV* (53) or the Diagnostic Interview for ADHD in adults (54) can be used for this purpose.

In a following step, disorder-relevant symptoms and their manifestation in individuals are assessed by using standardized methods for detailed assessment. For this purpose, there are several validated instruments available in different languages, such as Adult ADHD Self-Report Scale and the short version for screening, Wender-Utah-Rating-Scale for childhood symptoms, or Conners' or Brown's scale for current symptoms. The Conners' or Brown's scale is recommended to assess the magnitude of the impairments caused by ADHD. As they are normed (55), a severity classification (mild, moderate, and severe) can be conducted by comparing the results with the norm population. Alternatively, there are also batteries (e.g., Homburger ADHD scales for adults) available, which cover all relevant instruments (56).

Finally, a neuropsychological assessment provides relevant information about the general level of cognitive performance and existing neurocognitive deficits. Mainly neuropsychological tests aiming at attentional and executive functions as well as tests to measure various aspects of intelligence are used.

The decision for a therapy, respectively, or the choice of a specific treatment depends on the severity of the current situation, mental and social impairments, present comorbid disorders, the relevance of the symptoms in performing daily routine tasks, and existing resources (23). Ideally, the treatment should include several components, such as pharmacotherapy, cognitive behavioral therapy in individual or group format, psychoeducation, and peer support (57). Pharmacotherapy and behavioral therapy seem to have similar therapeutic effects on ADHD symptoms in adolescents (23). In adults with ADHD and SUD, a combination of pharmacotherapy and psychotherapy is recommended (22, 58, 59). When it comes to pharmacotherapy, however, treatment with stimulants is the therapy of first choice (2, 50).

### Methylphenidate

MPH is a centrally acting psychostimulant (60) that is subject to the narcotics law. It is approved for the treatment of ADHD in children from 6 years of age, adolescents, and adults, as well as for the treatment of sleep disorders (e.g., excessive daytime sleepiness, narcolepsy) (61). In addition, off-label use of MPH to treat depression is also practiced (61). There are various immediate- and sustained-release preparations of multiple brands available for oral or transdermal administration.

MPH unfolds its stimulant, indirectly sympathomimetic effects by inhibiting presynaptic reuptake of dopamine and noradrenaline (61). Unlike classical reuptake inhibitors, it also induces rapid and significant rises in striatal (62) and accumbal (63) dopamine efflux, which seems to play a key role for the therapeutic effect of MPH (11). MPH leads to an upregulation of the frontoparietal executive function network and the temporoparietal attentional network, which is associated with improved attention in children with ADHD and better inhibitory control in the PFC (64). The calming effect of MPH in patients with ADHD is most likely connected with the improvement of dopamine deficiency (65). MPH has a high affinity toward the DAT, which is comparable to that of cocaine (65). According to Heal (62), “MPH and cocaine act as ‘inverse agonists,' reversing the usual direction of dopamine transport by DAT” (66).

Neuropharmacobehavioral studies of injected MPH and cocaine revealed not only similarities, but also significant differences between the two substances (67–69). For both drugs, the fast uptake in the striatum paralleled the “high” experience but only for cocaine the decline in the “high” corresponded to the brain clearance rate. In contrast, for MPH, the “high” decreased as rapidly as for cocaine despite significant striatal binding of the drug, suggesting that acute tolerance to the reinforcing effects of MPH had occurred (67, 69). The slow brain clearance of MPH may therefore limit its misuse potential (70).

### Misuse Potential of MPH

The misuse liability of MPH is well-known. In the literature, mostly intravenous and nasal administrations, which led to substance- or administration-specific complications, are described as cases of misuse (70). Complications of such administrations, particularly intravenous, with crushed tablets include local or vascular infections, foreign body reactions, granulomas in the lungs, and pulmonary arterial hypertension due to the blockage of the lung circulation (71–73). Studies that investigated the narrow path between the therapeutic impact and the misuse-supporting reinforcing effect of MPH found strong hints for the euphoric potential of MPH in people with as well as without histories of SUD (74, 75). While a slow increase in serum concentrations shows therapeutic effects, a rapid and steep increase results in subjective reinforcing effects (76, 77). Therefore, a rapid onset of action and high concentrations in the body are essential to get the “high,” which can be achieved by intravenous or nasal administration or by the intake of very high doses (76). Recent data collected in a sample of patients in OAT suggest that the need for a rapid onset of action is an important reason for MPH misuse in this population (68). However, an analysis of two studies, which investigated the subjective effects of osmotic-release oral systems MPH (OROS-MPH) showed substance use severity did not significantly affect the euphoric effects of OROS-MPH (78).

### Misuse and Diversion of MPH in ADHD Patients

Misuse and diversion are inherent risks of prescribing controlled substances, and a substantial minority of patients with prescribed MPH will misuse their own prescription or divert their medications to others (75). In recent years, an increase of cases of misuse and diversion has been reported, which goes along with an increase of ADHD diagnoses and thus prescription of MPH (71). However, the frequency of misuse in people with ADHD is not clear. In a prospective cohort study with patients who received prescribed MPH, 22% of the patients with ADHD reported to have misused MPH at least once (79). In another survey of a specialized institution, 14% of the patients with ADHD reported to have misused MPH (predominantly nasal) or another prescribed stimulant (80). In a national US survey, 8.9% of the participants with a prescription for ADHD medication reported to have sold, traded, or given away their medication (81). Mostly older adolescents and adults who consume also other psychotropic substances or have other behavioral disorders are at risk of doing so (71, 79, 82). A recent study suggests that MPH may also be misused by a substantial proportion of methadone-maintained patients (83). A survey in France showed that ~46% of MPH acquisitions by persons with substance dependence or on OAT were illegal (84). However, the diversion/misuse of OROS-MPH seems not to be affected by the substance use severity of the patients (78).

## Discussion and Conclusion

### MPH Therapy for Patients With ADHD and SUD

It has not yet been clarified to what extent ADHD treatment with MPH contributes to the development of SUDs. Current evidence is based on secondary analyses as prospective studies, to our knowledge, are still missing. The existing results are inconsistent; however, they do not indicate that MPH contributes to the development of SUDs. On the contrary, treating ADHD patients with MPH may reduce their risk for SUD (77, 85, 86). According to a meta-analysis, ADHD patients treated with MPH in their childhood show a reduced risk of developing SUD by a factor of 1.9 compared to untreated patients (79). Studies using data from large databases and registries showed that medication treatment of ADHD, mostly with stimulants, was associated with a significantly reduced risk of SUD outcomes (87–89) and that within individuals this reduction in risk of SUD outcomes is related to periods of adherence to the medication prescribed (87, 89). However, stimulant medication was neither associated with an increased nor decreased risk for SUD diagnoses in another study (90). A meta-analysis with data from two of these studies (88, 89) showed that the risk of substance use outcomes was reduced; however, it did not attain statistical significance (91). Research among patients in OAT with ADHD suggests that a cotherapy with MPH not only reduces ADHD symptoms and improves psychosocial functioning but also does not worsen the course of substance use (92).

The question is whether adults with both disorders should be treated with MPH despite its misuse potential and the risks connected to it. In due consideration of the scientific literature, the benefits of ADHD treatment with MPH outweigh its risks; therefore, in our opinion, treatment with MPH should be considered as first-line treatment also in adults with ADHD and SUD. However, these risks should be seriously taken into account when planning the therapy. Furthermore, a reasonable therapeutic setting should be arranged with patients for the prescription of MPH. Additionally, patients should be closely monitored for possible side effects and misuse/diversion of MPH.

**Figure 1:**
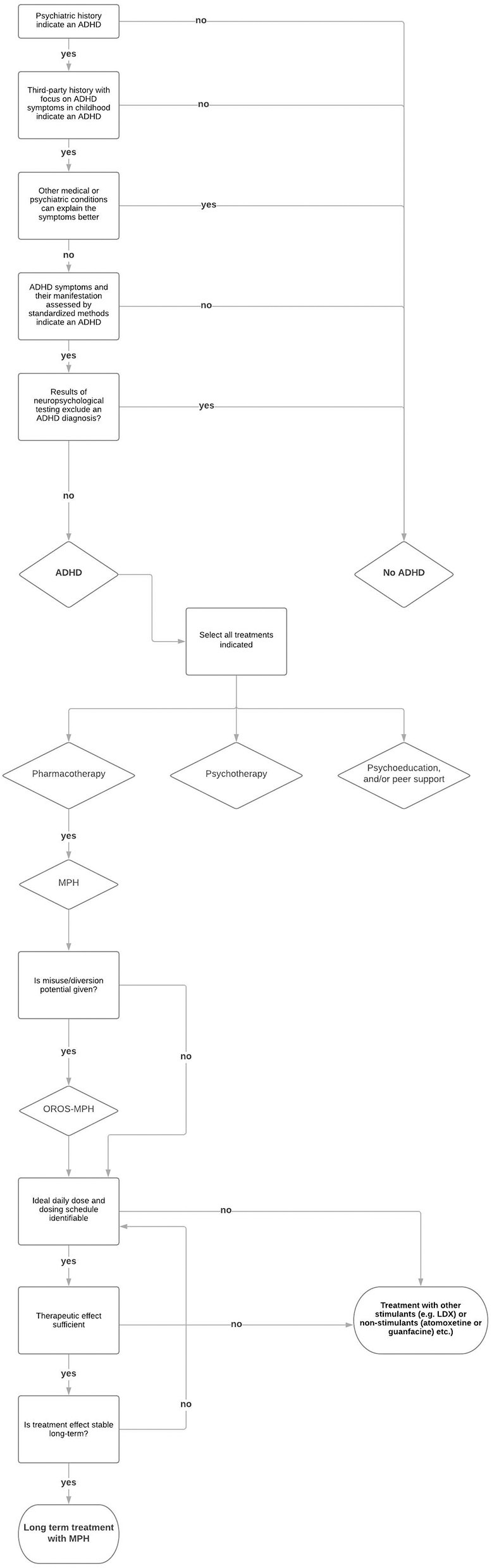
Diagnosis and pharmacotherapy for adult ADHD in patients with SUD.

In any case, a verified guideline-based diagnosis is the basic requirement for the treatment of ADHD. Insights from this diagnostic procedure can be used to plan the treatment and to determine the indication of therapy offers (93). The treatment of ADHD should be adjusted individually for patients with SUDs as for any other patient. The treatment and its duration can vary from patient to patient (94). The decision for a treatment depends on the current severity of ADHD, the psychosocial impairments, the existing comorbid disorders, and the relevance of symptoms in the context of existing resources (23, 95). The treatment with MPH is important, but often not enough. Regular psychotherapy sessions and specific interventions improve the success of the treatment significantly (96). Some patients need treatment with MPH only temporarily or periodically, e.g., when other compensation strategies are not sufficient anymore or when they need to face important private or job-related challenges. It is also relevant to find the adequate formulation, daily dose, and dosing schedule of MPH to achieve optimal therapeutic results, which should be verified regularly. However, the approved drugs and recommended dosages are often not enough to cover the therapeutic need (95). Because of the lower DAT availability and occupancy (97), MPH may have reduced efficacy in persons with SUD (98), especially in cocaine-dependent ADHD patients (98, 99). Lisdexamfetamine dimesylate (LDX), as the first long-acting prodrug stimulant for the treatment of ADHD (100), is possibly another safe and more potent option for adults with ADHD (101–103). LDX has low misuse potential due to its biological mechanism of enzymatic hydrolysis to obtain the active compound d-amphetamine (101). Also, non-stimulant α2-adrenoceptor agonists, such as guanfacine are an effective treatment option to be considered, but mainly in the combined hyperactive/impulsive-inattentive ADHD subgroup (66). Atomoxetine, a non-stimulant medication, might be effective for patients with ADHD and alcohol use disorder (104). However, studies determining the efficacy of these drugs in adults with ADHD and SUD are scarce. Figure 1 depicts a step-by-step procedure of diagnosing and treating SUD patients for ADHD.

In general, extended-release formulations of MPH are recommended for the treatment of adult ADHD (105), which may also prevent possible misuse of MPH, due to the lower misuse potential (77, 78, 106). Luo and Levin state that also from clinical experience, the misuse liability of long-acting formulations of prescribed psychostimulants is low (39). Other methods to prevent misuse are prescribing non-stimulant drugs (e.g., atomoxetine or guanfacine) or prescribing LDX. Regular clinical evaluation of MPH adherence and supervising the intake of medication are additional methods to prevent misuse (75). In cases of suspected diversion, regular rapid urine tests can be considered as an instrument to confirm the intake of medication by the patients. However, the rationale of these interventions must be clear and should be discussed with patients in detail as they will experience them as a violation of their autonomy, which could undermine the therapeutic relationship.

## Author Contributions

SC, JS, MV, and KD did the literature review and wrote the draft. MW and KD commented on the first draft. SB, MW, and KD commented on the second draft. All authors commented on the final manuscript, which was completed by SC, JS, MV, SB, MW, and KD.

## Conflict of Interest

The authors declare that the research was conducted in the absence of any commercial or financial relationships that could be construed as a potential conflict of interest.

## Abbreviations

ADHD: attention-deficit and hyperactivity disorder
SUD: substance use disorder
DAT: dopamine transporter
MPH: methylphenidate
OUD: opioid use disorder
OAT: opioid agonist treatment
VTA: ventral tegmental area
PFC: prefrontal cortex
ACC: anterior cingulate cortex
OROS-MPH: osmotic-release oral systems methylphenidate
LDX: Lisdexamfetamine dimesylate
ASRS v1.1: adult self-report scale.
